# Unveiling species diversity within early-diverging fungi from China XVI: Five new *Absidia* and a new *Gongronella* species (Cunninghamellaceae, Mucoromycota)

**DOI:** 10.3897/mycokeys.136.201393

**Published:** 2026-07-08

**Authors:** Fei Li, Ri-Peng Zhang, Jun-Nan Fang, Xiao-Jie Wang, Hong-Yu Zou, Shu-Ting Geng, Shi Wang, Ze-Min Fang, Xiao-Yong Liu

**Affiliations:** 1 College of Life Sciences, Shandong Normal University, Jinan 250358, China College of Life Sciences, Shandong Normal University Jinan China; 2 State Key Laboratory of Microbial Diversity and Innovative Utilization, Institute of Microbiology, Chinese Academy of Sciences, Beijing 100101, China State Key Laboratory of Microbial Diversity and Innovative Utilization, Institute of Microbiology, Chinese Academy of Sciences Beijing China; 3 School of Life Sciences and Medical Engineering, Anhui University, Hefei, Anhui 230601, China School of Life Sciences and Medical Engineering, Anhui University Hefei China

**Keywords:** Biodiversity, new taxa, phylogeny, taxonomy, terricolous fungi

## Abstract

The family Cunninghamellaceae demonstrates considerable species diversity among early-diverging fungi. Within this family, the genera *Absidia* and *Gongronella* are notably diverse and exhibit global distribution. Morphological and molecular analyses identified six new species from six provinces in China. Phylogenetic analyses using SSU, ITS, LSU, *TEF1α*, and *act* gene sequences indicate that *A. guangxiensis***sp. nov**. and *A. heilongjiangensis***sp. nov**. form a distinct clade; *A. hubeiensis***sp. nov**. is closely related to *A. cylindrospora*; and *A. longisporangiophora***sp. nov**. is sister to *A. myceliosa***sp. nov**. Morphologically, *Absidia guangxiensis* is defined by broad apophyses, *A. heilongjiangensis* by large projections, *A. hubeiensis* by large sporangia, *A. longisporangiophora* by elongated sporangiophores, and *A. myceliosa* by abundant mycelia. Phylogenetic analyses based on ITS, LSU, *TEF1α*, and *act* sequences demonstrate that *Gongronella globospora***sp. nov**. is closely related to *G. chlamydospora*, but is morphologically distinguished by globose sporangia. These findings expand the known biodiversity of Cunninghamellaceae in China and increase the global species count of *Absidia* and *Gongronella* to 71 and 34, respectively.

## Introduction

The family Cunninghamellaceae belongs to the Mucorales, Mucoromycetes, and Mucoromycota (https://www.indexfungorum.org/, accessed on April 29, 2026). It was first described by Benjamin (1959) and contains six accepted genera: *Absidia*, *Chlamydoabsidia*, *Cunninghamella*, *Gongronella*, *Halteromyces*, and *Hesseltinella*. The genera *Absidia* (with 66 accepted species) and *Gongronella* (with 33 accepted species) are the top two genera, accounting for the majority (59.6%) of this family. The genus *Absidia* was first proposed by Tieghem (1876), typified with *A. reflexa*. The genus *Gongronella* was first proposed by Ribaldi (1952), typified with *G. urceolifera*. Both genera have worldwide distribution. They are saprotrophic and isolated from various substrates, especially soils (Dong et al. 2019; Martins et al. 2020; Wang et al. 2023; Zhao et al. 2023; Wang et al. 2024; Ding et al. 2025b; Liu et al. 2026a).

Species of *Absidia* always develop sporangiophores from stolons, rhizoids at both ends of stolons, pyriform and deliquescent-walled apophysate sporangia, and subglobose columellae with apical projections (Tieghem 1876; Benny et al. 2001; Hoffmann et al. 2007). Members of *Gongronella* form coenocytic hyphae and swollen apophyses, a constriction between apophyses and sporangia, and sporangia for asexual reproduction (Ribaldi 1952; Ellis and Hesseltine 1964; Doilom et al. 2020; Martins et al. 2020).

These two genera have significant practical application value. The genus *Absidia* produces fatty acids, chitin, α-galactosidase, and other metabolites serving industrial and medicinal fields (Kaczmarek et al. 2019; Chen et al. 2020); it is also involved in wine fermentation (Zhao et al. 2022) and the biotransformation of various natural products (Kristanti et al. 2016; Zhao et al. 2022). The genus *Gongronella* similarly secretes a variety of metabolites, including chitosanases used in the chitosan industry (Tan et al. 1996; Babu et al. 2015); its activities also include solubilizing phosphate, degrading the fungicide metalaxyl (Doilom et al. 2020; Martins et al. 2020), inducing laccase production (Wei et al. 2010), and promoting plant growth and development by providing phosphorus (Wang et al. 2024; Fang et al. 2025, 2026).

In recent years, extensive taxonomic studies on *Absidia* and *Gongronella* in China have led to the description of numerous new species. For *Absidia*, Zhao et al. (2023) described 15 new species, followed by Luo et al. (2024) with one, Tao et al. (2024) with three, Ding et al. (2025b) with four, Ji et al. (2025b) with five, Wang et al. (2025) with four, and Liu et al. (2026a) with four (Zhao et al. 2023; Luo et al. 2024; Tao et al. 2024; Ding et al. 2025b; Ji et al. 2025b; Wang et al. 2025; Liu et al. 2026a). For *Gongronella*, Wang et al. (2023) described three new species, followed by Zhao et al. (2023) with two, Wang et al. (2024) with six, Wei et al. (2024) with one, and Liu et al. (2025) with two (Wang et al. 2023; Zhao et al. 2023; Wang et al. 2024; Wei et al. 2024; Liu et al. 2025).

In the present study, six new species belonging to the family Cunninghamellaceae are proposed based on morphological and phylogenetic analyses. They were isolated from four distinct locations across China. This is the sixteenth study in a series on the biodiversity of early-diverging fungi in China (Tao et al. 2024; Wang et al. 2024; Zhao et al. 2024; Ding et al. 2025a, 2025b, 2025c; Ji et al. 2025a, 2025b, 2025c; Jiang et al. 2025; Wang et al. 2025; Liu et al. 2026a, 2026b).

## Materials and methods

### Collection and isolation

Soil samples were collected in 2024 from six provinces in China. These are Anhui Province, Fujian Province, Guangdong Province, Guangxi Zhuang Autonomous Region, Heilongjiang Province, and Hubei Province. The collection methods followed the protocols of Liu et al. (2019) and Zou et al. (2022). Each sample was placed in a sealed plastic bag and assigned a specific sample number. Detailed records were kept for each sample, including the collector, date, and altitude, latitude, and longitude (Rathnayaka et al. 2025). After collection, the samples were delivered to the laboratory and stored at 4 °C until isolation procedures were conducted.

Fungal isolation used the spread and streak plate methods (Senanayake et al. 2020). Initially, soil samples were spread onto Rose Bengal Chloramphenicol Agar (RBC) (Corry et al. 2003; Wang et al. 2024) and cultured at 25 °Cin the dark. Single spores from fungal colonies were picked under a stereomicroscope (Olympus SZX10, Tokyo, Japan) and transferred onto Potato Dextrose Agar (PDA: glucose 20.00 g/L, potato extract 200.00 g/L, agar 17.00 g/L, and pH 7.0) to grow into pure colonies. Pure strains were preserved in 10% sterilized glycerol at 4 °C.

The living cultures were preserved at Shandong Normal University (**XG**) and China General Microbiological Culture Collection Center, Beijing, China (**CGMCC**). The dried holotypes were preserved at the Herbarium Mycologicum Academiae Sinicae in Beijing, China (**HMAS**).

### Morphology

The pure colony on PDA was photographed with a digital camera (Canon PowerShot G7 X, Canon, Tokyo, Japan). Microscopic morphological structures were observed and recorded with an optical microscope (Olympus BX53, Tokyo, Japan). The size of fungal structures was measured using Digimizer software v5.6.0 (https://www.digimizer.com, accessed on April 24, 2026). The photographs were assembled into composite figures using Adobe Photoshop CC 2019 (https://adobe.com/products/photoshop, accessed on April 24, 2026).

### DNA extraction, PCR amplification, and sequencing

Five genomic loci, small subunit (SSU), internal transcribed spacer (ITS), large subunit of ribosomal DNA (LSU rDNA), translation elongation factor 1 α (*TEF1α*), and actin (*act*) were used for *Absidia* identification. ITS, LSU, *act*, and *TEF1α* were used for *Gongronella* identification. Genomic DNAs were extracted from fungal mycelia using GOMag^TM^ Rapid Plant DNA Kit (GO-GPLF-400). These five loci ITS, LSU, *TEF1α*, *act*, and SSU were amplified using the primer pairs ITS5/ITS4, LR0R/LR5, EF1-983F/TEF1LLErev, Act-1/Act-4R, and NS1/NS4, respectively. The 25 µL PCR mixture consisted of 9.5 µL of double-distilled water (ddH_2_O), 1 µL of forward primer (10 µM, TsingKe, Beijing, China), 1 µL of reverse primer (10 µM, TsingKe, Beijing, China), 1 µL of template DNA, and 12.5 µL of 2× Taq Master Mix (Dye Plus) (Vazyme, Pack NO.:037E3231CD). Genomic loci, PCR primers and sequences, and PCR programs used were summarized in Table 1. Sanger sequencing was conducted by Sangon Biotech (Shanghai, China) Co., Ltd. Consensus sequences were generated with Geneious Prime 2025.0.2 (https://www.geneious.com, accessed on April 23, 2026).

**Table 1.** Genomic loci, PCR primers and sequences, and PCR programs used in this article.

**Genomic loci**	**PCR primers**	**Primer sequence (5’–3’)**	**PCR programs**	**References**
ITS	ITS5	GGA AGT AAA AGT CGT AAC AAG G	95 °C 5 min; (95 °C 30 s, 55 °C 30 s, 72 °C 60 s) × 35 cycles; 72 °C 10 min	(White et al. 1990)
	ITS4	TCC TCC GCT TAT TGA TAT GC		
LSU	LR0R	GTA CCC GCT GAA CTT AAG C	95 °C 5 min; (94 °C 30 s, 52 °C 45 s, 72 °C 90 s) × 30 cycles; 72 °C 10 min	(Hurdeal et al. 2023)
	LR5	TCC TGA GGG AAA CTT CG		
*TEF1α*	EF1-983F	GCY CCY GGH CAY CGT GAY TTY AT	95 °C 5 min; (95 °C 30 s, 55 °C 1 min, 72 °C 1 min) × 30 cycles; 72 °C 10 min	(Jaklitsch et al. 2005; Rehner and Buckley 2005)
	TEF1LLErev	AAC TTG CAG GCA ATG TGG		
*act*	Act-1	TGG GAC GAT ATG GAI AAI ATC TGG CA	94 °C 5 min; (94 °C 30 s, 52 °C 60 s, 72 °C 60 s) × 35 cycles; 72 °C 10 min	(Voigt and Wöstemeyer 2000)
	Act-4R	TC ITC GTA TIC TIG CTI IGA IAT CCA CA T		
SSU	NS1	GTA GTC ATA TGC TTG TCT C C	95 °C 5 min; (94 °C 1 min, 54 °C 50 s, 72 °C 1 min) × 37 cycles; 72 °C 10 min	(White et al. 1990)
	NS4	CTT CCG TCA ATT CCT TTA AG		

### Phylogenetic analyses

All strains and sequences appearing in this study were chosen with references to the most recent literature on *Absidia* and *Gongronella* (Wang et al. 2024; Tan et al. 2025; Liu et al. 2026a). The GenBank accession numbers of all sequences used in the article were summarized in Suppl. material 1 and can be obtained from the National Center for Biotechnology Information (NCBI) (https://www.ncbi.nlm.nih.gov/, accessed on May 06, 2026). The obtained original sequences of the new species were assembled and adjusted by Geneious Prime 2025.0.2 (https://www.geneious.com, accessed on April 23, 2026). Preliminary species identifications were performed using the BLAST algorithm at NCBI (https://blast.ncbi.nlm.nih.gov/Blast.cgi, accessed on April 23, 2026). Sequences were aligned with MAFFT (https://mafft.cbrc.jp/alignment/software/, accessed on April 23, 2026), and trimmed by TrimAl (Galaxy Version 1.5.1+galaxy0) (Capella-Gutiérrez et al. 2009). The final datasets ITS-LSU-*TEF1α*-*act*-SSU for *Absidia* and ITS-LSU-*act*-*TEF1α* for *Gongronella* were concatenated in MEGA v.7.0 (Kumar et al. 2016). Phylogenetic analyses of the genera *Absidia* and *Gongronella* were conducted using two algorithms, Maximum Likelihood (ML) and Bayesian Inference (BI). ML analyses were conducted on the CIPRES Science Gateway V.3.3 (https://www.phylo.org/, accessed on May 06, 2026) by the method of RAxML-HPC 2 on ACCESS (8.2.12) with 1,000 bootstrap replicates under the GTR model (Miller et al. 2010; Stamatakis 2014). BI analyses were carried out using the GTR+I+G model, sampling every 1,000 generations, with twelve cold Markov chains running simultaneously for 50 million generations. The original phylogenetic tree files were visualized on the Interactive Tree of Life (iTOL) Version 7.2 (https://itol.embl.de, accessed on April 23, 2026). Phylogenetic trees were adjusted and beautified by Adobe Illustrator CC 2019 (https://adobe.com/products/illustrator, accessed on May 06, 2026).

## Results

### Molecular phylogeny

**Figure 1.**
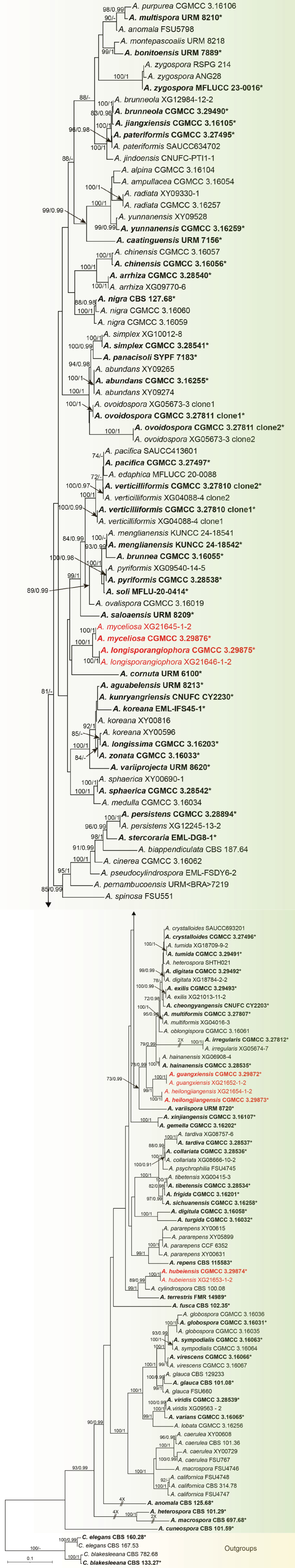
Phylogenetic tree of *Absidia* based on ITS-LSU-*TEF1α*-*act*-SSU sequences, with *Cunninghamella elegans* and *C. blakesleeana* as outgroups. The concatenated dataset comprises 152 strains and 4,963 characters. The best RAxML tree had a final likelihood value of –40469.077957. The evolutionary model GTR+I+G is applied for all genes. The matrix contained 2,140 distinct alignment patterns, with 51% of characters being completely undetermined or missing. Maximum Likelihood Bootstrap Values (left, MLBV ≥ 70%) and Bayesian Inference Posterior Probability (right, BIPP ≥ 0.90) are shown on the nodes, divided by a slash “/”. MLBV < 70% and BIPP < 0.90 are represented by an en dash “–”. The ten newly proposed strains are indicated in bold red. Bold strains marked with an asterisk “*” represent ex-types or ex-holotypes. Branches shortened to fit the page are represented by double slashes “//” and the number of folds “×”. The scale at the bottom left indicates 0.1 substitutions per site.

Molecular phylogenetic analyses of the genus *Absidia* were based on a dataset comprising 152 strains, including 148 *Absidia* strains as in-groups and 4 *Cunninghamella* strains as out-groups. The out-group species are *C. blakesleeana* and *C. elegans*. The generated ITS-LSU-*TEF1α*-*act*-SSU sequences consisted of 4,963 characters: 1–402 (ITS), 403–1631 (LSU), 1632–3045 (*TEF1α*), 3046–3849 (*act*), and 3850–4963 (SSU). The sequence matrix comprised 3,157 constant, 1,518 parsimony-informative, and 288 parsimony-uninformative characters. The maximum likelihood (ML) tree (Fig. 1) and the Bayesian tree inferred the evolutionary relationships of *Absidia*.

**Figure 2.**
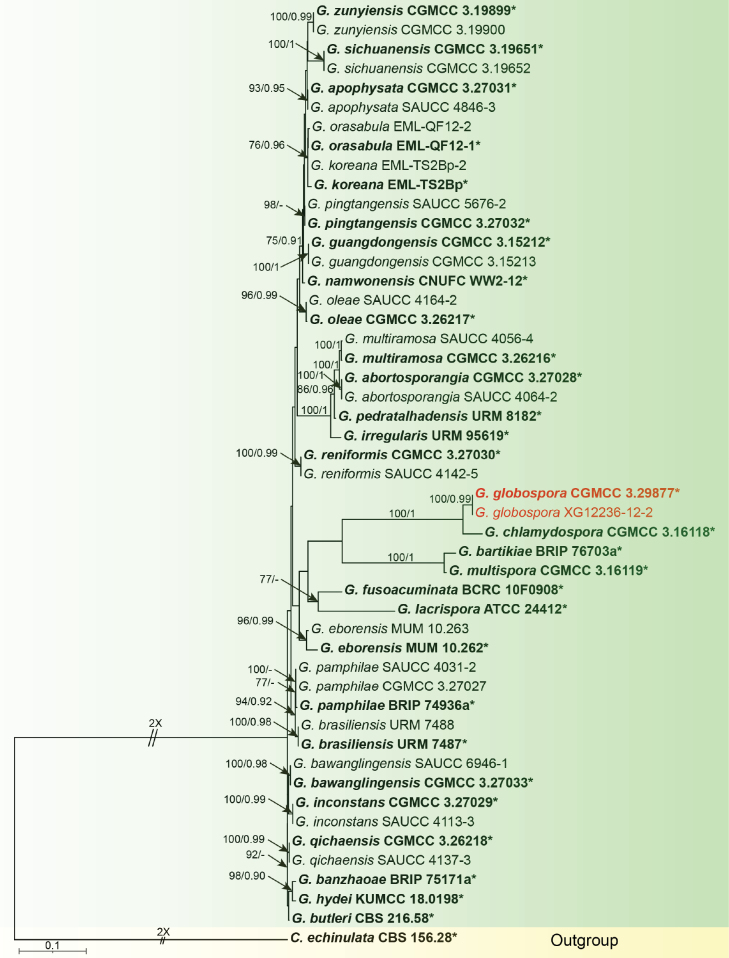
Phylogenetic tree of *Gongronella* based on ITS-LSU-*act*-*TEF1α* sequences, with *Cunninghamella echinulata* as an outgroup. The concatenated dataset comprises 49 strains and 3,052 characters. The best RAxML tree has a final likelihood value of –12535.903111. The evolutionary model GTR+I+G is applied for all genes. The matrix contained 858 distinct alignment patterns, with 30% of characters and gaps completely undetermined. Maximum Likelihood Bootstrap Values (left, MLBV ≥ 70%) and Bayesian Inference Posterior Probability (right, BIPP ≥ 0.90) are shown on the nodes, divided by a slash “/”. MLBV < 70% and BIPP < 0.90 are represented by an en dash “–”. The two newly proposed strains are indicated in bold red. Bold strains marked with an asterisk “*” represent ex-types or ex-holotypes. Branches shortened to fit the page are represented by double slashes “//” and the number of folds “×”. The scale at the bottom left indicates 0.1 substitutions per site.

Phylogenetic analyses of the genus *Gongronella* were based on a dataset comprising 49 strains, including 48 *Gongronella* strains as ingroups and *Cunninghamella echinulata* as the outgroup. The generated ITS-LSU-*act*-*TEF1α* sequences consisted of 3052 characters: 1–670 (ITS), 671–2084 (LSU), 2085–2849 (*act*), and 2850–3052 (*TEF1α*). The sequence matrix comprised 2042 constant, 688 parsimony-informative, and 322 parsimony-uninformative characters. The maximum likelihood (ML) tree (Fig. 2) and the Bayesian tree reconstructed the evolutionary relationships of *Gongronella*.

### Taxonomy

#### Absidia guangxiensis

Taxon classificationFungiMucoralesCunninghamellaceae

F. Li, H. Zhao & X.Y. Liu sp. nov.

E7F7F5DB-4FC3-53F4-88C1-571873563DC4

Fungal Names: FN 573945

Fig. 3

##### Etymology.

The epithet *guangxiensis* (Lat.) refers to the type locality, Guangxi Zhuang Autonomous Region.

**Figure 3.**
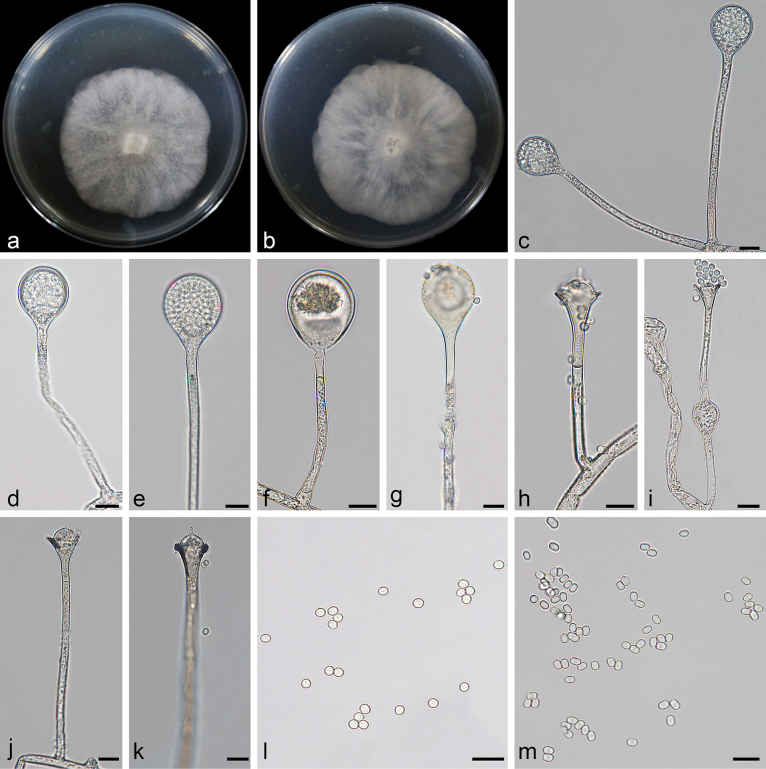
Morphologies of *Absidia guangxiensis* (HMAS 354597, holotype). **a, b**. Colonies on PDA (**a**. Obverse; **b**. Reverse); **c**. Whorled sporangiophores; **d, e**. Sporangiophores with sporangia; **f, g**. Sterile sporangia; **h**. Columellae with apophyses; **i**. Swellings below apophyses; **j, k**. Columellae with projections and collars; **l, m**. Sporangiospores. Scale bars: 10 µm (**c–m**).

##### Holotype.

HMAS 354597.

##### Description.

Colonies on PDA at 25 °C for 4 days, growing moderately, attaining 57–60 mm in diameter, showing an average growth rate of approximately 14.3–15.0 mm/d, initially white, becoming light yellow with age, fluffy, concentrically zonate with ring, and radially in reverse. Hyphae branched, subhyaline, septate, and 4.1–8.8 µm in diameter. Stolons subhyaline, gradually becoming brownish when mature, smooth, branched, septate, and 4.2–11.5 µm in diameter. Rhizoids unobserved. Sporangiophores arising from stolons, smooth, erect or bent, unbranched or simply branched, monopodial or sympodial, some 2–5 in whorls, subhyaline, dark brownish when mature, often with a septum 2.7–5.5 µm below the apophysis, 22.7–132.3 µm long, and 3.4–4.5 µm wide. Sporangia pyriform, smooth, subhyaline, deliquescent-walled, multi-spored, 17.8–30.0 µm long, and 13.2–34.8 µm wide. Sterile sporangia present. Apophyses distinct, subhyaline, 3.6–14.4 µm high, gradually widening from the base to the top, 2.3–6.1 µm wide at the base, and 5.6–18.8 µm wide at the top. Collars present. Columellae subglobose, subhyaline, 7.6–11.1 µm long, and 11.6–16.1 µm wide. Projections present, single, pacifier-like, subhyaline, 1.5–3.4 µm long, and 1.5–1.8 µm wide. Sporangiospores cylindrical or globose, sage green, smooth, 2.9–5.3 µm long, and 2.6–3.1 µm wide. Chlamydospores absent. Zygospores unobserved.

##### Materials examined.

China • Guangxi Zhuang Autonomous Region, Fangchenggang City, Shangsi County, Shiwandashan National Forest Park (21°54.35'N, 107°54.22'E), in the soil, 24 November 2024, Heng Zhao (holotype HMAS 354597, ex-holotype living culture CGMCC 3.29872 = XG21652-1-1); China • Anhui Province, Lu’an City, Huoshan County (31°8.12'N, 115°54.55'E), in the soil, 26 October 2025, Xin-Yu Ji, living culture XG24056-1; China • Guangdong Province, Zhaoqing City, Dinghu District, Dinghu Mountain Scenic Area, the Feiqian Bridge at Qingyun Temple (23°10.58'N, 112°32.24'E), in the soil, 27 November 2025, Hong-Yu Zou, living culture XG24704-1.

##### GenBank accession numbers.

ITSPZ378773; LSUPZ381883; SSUPZ385211; *act*PZ397471; *TEF1α*PZ414691.

##### Notes.

In the phylogenetic analyses, based on the concatenated ITS-LSU-*TEF1α*-*act*-SSU sequences, the newly presented strains of *Absidia guangxiensis* sp. nov. are closely related to another new species, *A. heilongjiangensis* (Fig. 1: MLBS = 99%, BIPP = 1). The pairwise identity values between *A. guangxiensis* and *A. heilongjiangensis* are 91.5% for ITS, 100% for LSU, 91.8% for *TEF1α*, 93.3% for *act*, and 92.6% for SSU. Morphologically, many distinct divergences are there between these two new species: faster growth rate (14.3–15.0 mm/d vs. 11.8–12.5 mm/d), wider hyphae (4.1–8.8 µm vs. 3.6–6.5 µm), wider stolons (4.2–11.5 µm vs. 3.4–9.2 µm), longer and wider sporangiophores (22.7–132.3 × 3.4–4.5 µm vs. 26.8–125.4 × 2.5–3.8 µm), wider apophyses (5.6–18.8 µm vs. 5.5–9.2 µm), smaller projections (1.5–3.4 × 1.5–1.8 µm vs. 4.8–4.9 × 1.4–2.3 µm), and larger sporangiospores (2.9–5.3 × 2.6–3.1 µm vs. 2.1–3.6 × 1.7–2.3 µm).

#### Absidia heilongjiangensis

Taxon classificationFungiMucoralesCunninghamellaceae

F. Li, H. Zhao & X.Y. Liu sp. nov.

DC118105-F069-5394-BF79-272193B5C3F6

Fungal Names: FN 573946

Fig. 4

##### Etymology.

The epithet *heilongjiangensis* (Lat.) refers to the type locality, Heilongjiang Province.

**Figure 4.**
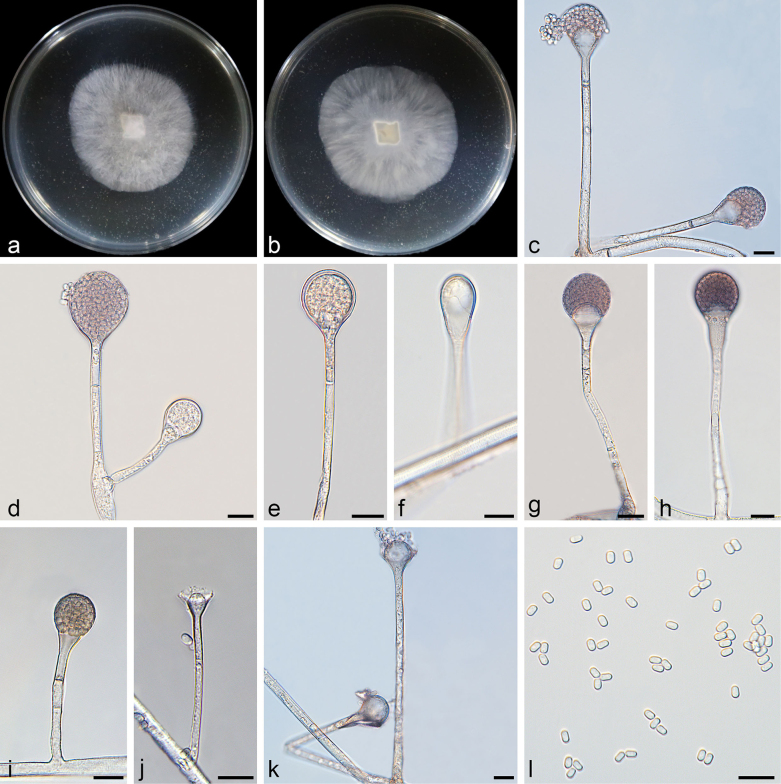
Morphologies of *Absidia heilongjiangensis* (HMAS 354598, holotype). **a, b**. Colonies on PDA (**a**. Obverse; **b**. Reverse); **c**. Whorled sporangiophores; **d**. Branched sporangiophores with sporangia; **e**. Sporangiophores with sporangia; **f**. Sterile sporangia; **g–i**. Columellae with apophyses; **j, k**. Columellae with projections and collars; **l**. Sporangiospores. Scale bars: 10 µm (**c–l**).

##### Holotype.

HMAS 354598.

##### Description.

Colonies on PDA at 25 °C for 4 days, growing moderately, attaining 47–50 mm in diameter, showing an average growth rate of approximately 11.8–12.5 mm/d, white, velvety, concentrically zonate with a ring, and radially in reverse. Hyphae branched, subhyaline, and 3.6–6.5 µm in diameter. Stolons subhyaline to hyaline, gradually becoming brownish when mature, smooth, branched, septate, and 3.4–9.2 µm in diameter. Rhizoids unobserved. Sporangiophores arising from stolons, smooth, erect or recumbent, unbranched or simply branched, monopodial or sympodial, some 2–3 in whorls, hyaline or subhyaline, brownish when mature, often with a septum 2.6–4.8 µm below the apophysis, 26.8–125.4 µm long, and 2.5–3.8 µm wide. Sporangia pyriform, smooth, subhyaline, deliquescent-walled, multi-spored, 15.6–31.5 µm long, and 9.7–25.6 µm wide. Sterile sporangia present. Apophyses distinct, subhyaline, 3.4–12.2 µm high, gradually widening from the base to the top, 1.8–4.7 µm wide at the base, and 5.5–9.2 µm wide at the top. Collars present. Columellae subglobose, hyaline, 8.5–12.8 µm long, and 11.0–15.9 µm wide. Projections present, single, pacifier-like, subhyaline, 4.8–4.9 µm long, and 1.4–2.3 µm wide. Sporangiospores usually cylindrical, sage green, smooth, 2.1–3.6 µm long, and 1.7–2.3 µm wide. Chlamydospores absent. Zygospores unobserved.

##### Materials examined.

China • Heilongjiang Province, Harbin City, Acheng District (45°12.77'N, 127°16.75'E), in the soil, 05 August 2024, Heng Zhao (holotype HMAS 354598, ex-holotype living culture CGMCC 3.29873 = XG21654-1-1).

##### GenBank accession numbers.

ITSPZ378775; LSUPZ381885; SSUPZ385213; *act*PZ397475; *TEF1α*PZ414695.

##### Notes.

In the phylogenetic analyses, based on the concatenated ITS-LSU-*TEF1α*-*act*-SSU sequences, the newly identified strains of *Absidia heilongjiangensis* sp. nov. are closely related to the aforementioned *A. guangxiensis* (Fig. 1: MLBS = 99%, BIPP = 1). The pairwise identity values between *A. heilongjiangensis* and *A. guangxiensis* are 91.5% for ITS, 100% for LSU, 91.8% for *TEF1α*, 93.3% for *act*, and 92.6% for SSU. The previous notes of *A. guangxiensis* provide the morphological differences between the two new species.

#### Absidia hubeiensis

Taxon classificationFungiMucoralesCunninghamellaceae

F. Li, H. Zhao & X.Y. Liu sp. nov.

287AEC09-9A03-511C-900A-EED429385E79

Fungal Names: FN 573947

Fig. 5

##### Etymology.

The epithet *hubeiensis* (Lat.) refers to the type locality, Hubei Province.

**Figure 5.**
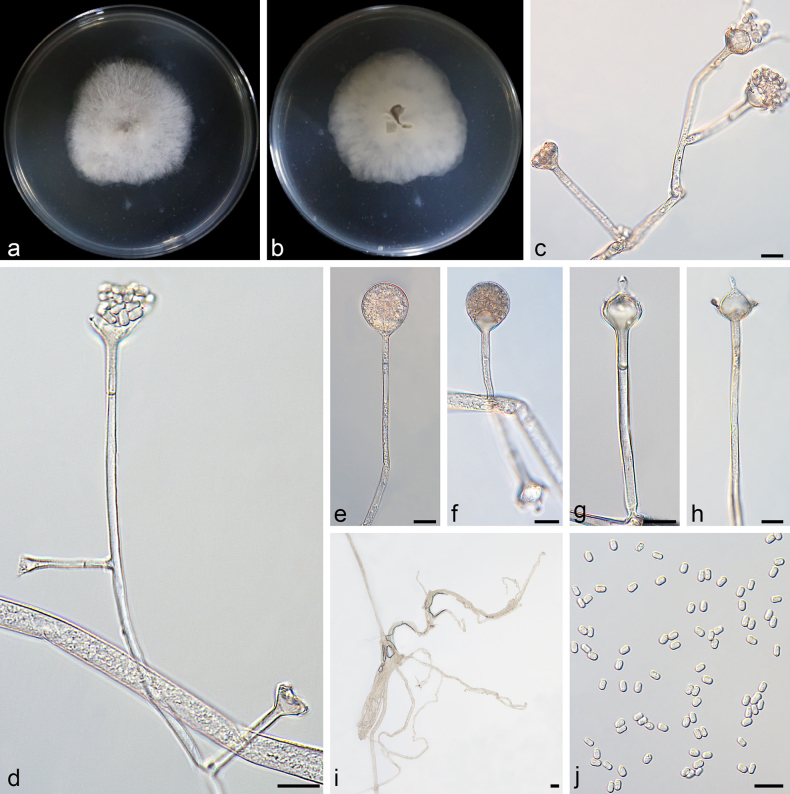
Morphologies of *Absidia hubeiensis* (HMAS 354599, holotype). **a, b**. Colonies on PDA (**a**. Obverse; **b**. Reverse); **c, d**. Branched sporangiophores with columellae and apophyses; **e, f**. Sporangiophores with sporangia; **g, h**. Columellae with projections and collars **i**. Rhizoids; **j**. Sporangiospores. Scale bars: 10 µm (**c–j**).

##### Holotype.

HMAS 354599.

##### Description.

Colonies on PDA at 25 °C for 4 days, growing moderately, attaining 43–50 mm in diameter, showing an average growth rate of approximately 10.8–12.5 mm/d, snowy white, velvety, and with a wave-like edge on the reverse. Hyphae branched, subhyaline, septate, and 5.4–16.0 µm in diameter. Stolons subhyaline to hyaline, gradually becoming brownish when mature, smooth, branched, some septate, and 2.5–9.3 µm in diameter. Rhizoids present, root-like, branched, and subhyaline. Sporangiophores arising from stolons, smooth, erect or bent, unbranched or simply branched, monopodial or sympodial, hyaline or subhyaline, brownish when mature, often with a septum 1.6–3.7 µm below the apophysis, 18.8–148.7 µm long, and 1.5–4.5 µm wide. Sporangia pyriform, smooth, subhyaline, deliquescent-walled, multi-spored, 8.6–27.2 µm long, and 9.0–28.0 µm wide. Apophyses absent or distinct, subhyaline, 2.1–9.3 µm high, gradually widening from the base to the top, 3.9–5.4 µm wide at the base, and 8.6–20.8 µm wide at the top. Collars present. Columellae globose, hyaline, 7.5–13.7 µm long, and 9.6–14.1 µm wide. Projections present, single, pacifier-like, subhyaline, 3.9–6.8 µm long, and 1.5–3.5 µm wide. Sporangiospores usually cylindrical, sage green, smooth, 3.1–4.4 µm long, and 1.7–2.8 µm wide. Chlamydospores absent. Zygospores unobserved.

##### Materials examined.

China • Hubei Province, Huanggang City, Xishui County, Sanjiao Mountain National Forest Park (30°17.50'N, 115°19.40'E), in the soil, 15 March 2024, Heng Zhao (holotype HMAS 354599, ex-holotype living culture CGMCC 3.29874 = XG21653-1-1).

##### GenBank accession numbers.

ITSPZ378777; LSUPZ381887; SSUPZ385215; *act*PZ397473; *TEF1α*PZ414693.

##### Notes.

In the phylogenetic analyses, based on the concatenated ITS-LSU-*TEF1α*-*act*-SSU sequences, the newly identified strains of *Absidia hubeiensis* are closely allied to *A. cylindrospora* (Fig. 1: MLBS = 89%, BIPP = 0.99). The pairwise identity values between *A. hubeiensis* and *A. cylindrospora* are 89.5% for ITS, 99.3% for LSU. The values of *TEF1α*, *act*, and SSU are not available. Morphologically, differences between *A. hubeiensis* and *A. cylindrospora* (Ho et al. 2004) include slower growth rate (10.8–12.5 mm/d vs. 12.9 mm/d), shorter sporangiophores (18.8–148.7 × 1.5–4.5 µm vs. 130.0–230.0 × 2.5–4.5 µm), larger sporangia (8.6–27.2 × 9.0–28.0 µm vs. 15.1–22.5 × 17.5–25.0 µm), smaller columellae (7.5–13.7 × 9.6–14.1 µm vs. 12.2–21.2 × 12.3–15.1 µm), and larger sporangiospores (3.1–4.4 × 1.7–2.8 µm vs. 3.5–4.1 × 1.1–2.0 µm).

#### Absidia longisporangiophora

Taxon classificationFungiMucoralesCunninghamellaceae

F. Li, H. Zhao & X.Y. Liu sp. nov.

FDAF6FBE-225B-54E7-9AE1-41B039D3CA1C

Fungal Names: FN 573948

Fig. 6

##### Etymology.

The epithet *longisporangiophora* (Lat.), refers to the long sporangiophores.

**Figure 6.**
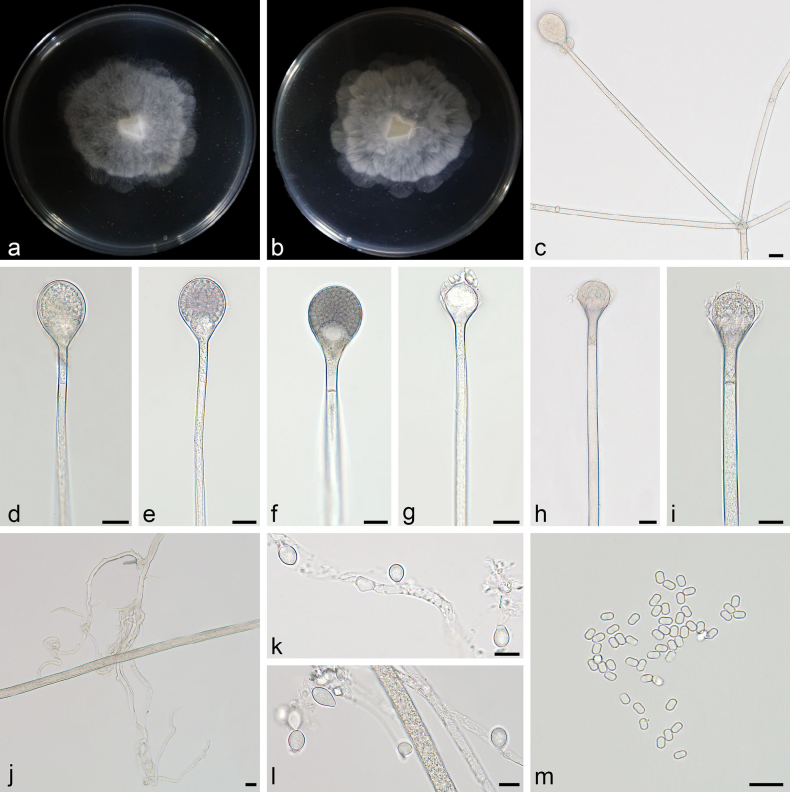
Morphologies of *Absidia longisporangiophora* (HMAS 354600, holotype). **a, b**. Colonies on PDA (**a**. Obverse; **b**. Reverse); **c**. Whorled sporangiophores with sporangia; **d–f**. Sporangiophores with sporangia and columellae; **g, h**. Columellae with apophyses and collars; **i**. Columellae with collars and projections; **j**. Rhizoids; **k, l**. Chlamydospores; **m**. Sporangiospores. Scale bars: 10 µm (**c–m**).

##### Holotype.

HMAS 354600.

##### Description.

Colonies on PDA at 25 °C for 4 days, growing moderately, attaining 47–50 mm in diameter, showing an average growth rate of approximately 11.8–12.5 mm/d, white, velvety, irregularly and concentrically zonate with a ring, and flower-shaped in reverse. Hyphae branched, subhyaline, light grayish, septate, and 3.4–12.6 µm in diameter. Stolons subhyaline, gradually becoming yellowish when mature, smooth, branched, and 4.6–13.0 µm in diameter. Rhizoids abundant, root-like, branched, and subhyaline. Sporangiophores arising from stolons, smooth, mostly erect, unbranched or simply branched, monopodial or sympodial, some 3–4 in whorls, mostly subhyaline, brownish when mature, often with a septum 3.5–5.6 µm below the apophysis, 145.0–372.7 µm long, and 4.0–7.7 µm wide. Sporangia pyriform, smooth, subhyaline, dark purple when mature, deliquescent-walled, multi-spored, 17.5–30.6 µm long, and 19.4–23.8 µm wide. Apophyses distinct, subhyaline, 5.4–9.7 µm high, gradually widening from the base to the top, 4.0–6.6 µm wide at the base, and 10.1–13.8 µm wide at the top. Collars present. Columellae subglobose, subhyaline, 8.4–14.8 µm long, and 13.0–19.1 µm wide. Projections present, single, pacifier-like, subhyaline, 3.5–4.5 µm long, and 1.6–1.9 µm wide. Sporangiospores usually cylindrical, sage green, smooth, 3.4–3.8 µm long, and 2.1–2.4 µm wide. Chlamydospores abundant, terminal, and thick-walled. Zygospores not observed.

##### Materials examined.

China • Guangxi Zhuang Autonomous Region, Chongzuo City, Jiangzhou District, Provincial Highway 313, Chongzuo Ecological Park (22°16.64'N, 107°30.77'E), in the soil, 28 November 2024, Heng Zhao (holotype HMAS 354600, ex-holotype living culture CGMCC 3.29875 = XG21646-1-1).

##### GenBank accession numbers.

ITSPZ378779; LSUPZ381889; SSUPZ385217; *act*PZ397469; *TEF1α*PZ414689.

##### Notes.

In the phylogenetic analyses, based on the concatenated ITS-LSU-*TEF1α*-*act*-SSU sequences, the newly identified strains of *Absidia longisporangiophora* are intimately allied to *A. myceliosa* (Fig. 1: MLBS = 100%, BIPP = 1), which is also described in the current study. The pairwise identity values between *A. longisporangiophora* and *A. myceliosa* are 95.6% for ITS, 98.9% for LSU, 98.6% for *TEF1α*, 99.4% for *act*, and 99.8% for SSU. Morphologically, they are obviously different: growth rate (11.8–12.5 mm/d vs. 6.8–7.2 mm/d), hyphae (3.4–12.6 µm vs. 3.8–7.7 µm), stolons (4.6–13.0 µm vs. 2.9–6.5 µm), sporangiophores (145.0–372.7 × 4.0–7.7 µm vs. 35.9–171.5 × 2.5–4.4 µm), sporangia (17.5–30.6 × 19.4–23.8 µm vs. 18.5–18.9 × 15.3–17.4 µm), apophyses (10.1–13.8 µm vs. 6.0–9.3 µm), columellae (8.4–14.8 × 13.0–19.1 µm vs. 6.5–11.5 × 6.6–10.8 µm) and projections (3.5–4.5 × 1.6–1.9 µm vs. 3.9–9.5 × 1.0–2.4 µm).

#### Absidia myceliosa

Taxon classificationFungiMucoralesCunninghamellaceae

F. Li, H. Zhao & X.Y. Liu sp. nov.

C837560A-C81D-59DE-BCEB-5839B628747F

Fungal Names: FN 573949

Fig. 7

##### Etymology.

The epithet *myceliosa* (Lat.), referring to the luxuriant mycelia.

**Figure 7.**
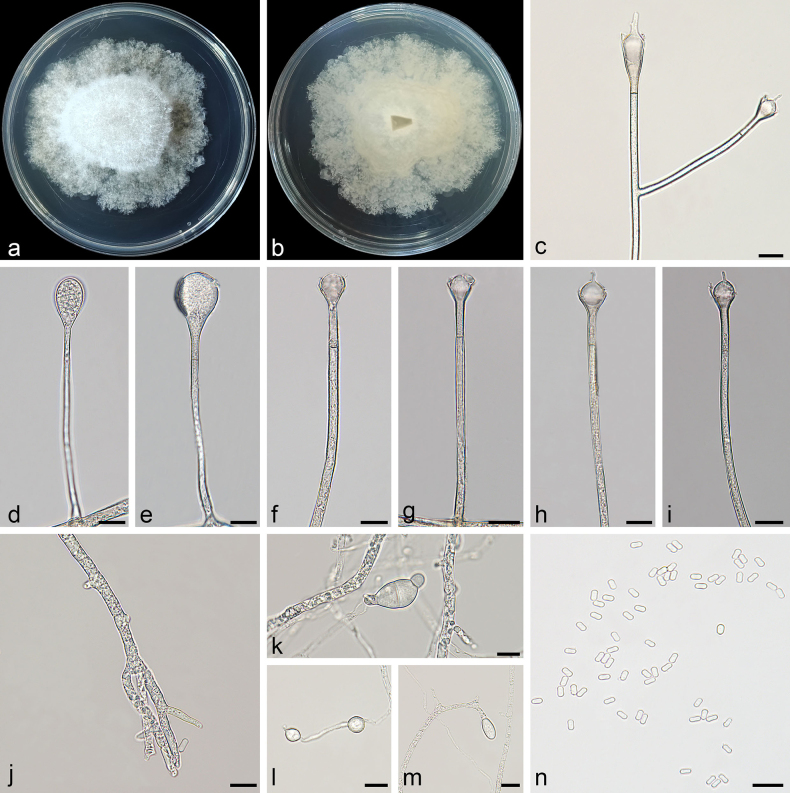
Morphologies of *Absidia myceliosa* (HMAS 354601, holotype). **a, b**. Colonies on PDA (**a**. Obverse; **b**. Reverse); **c**. Branched sporangiophores with columellae and projections; **d, e**. Sporangiophores with sporangia; **f, g**. Columellae with apophyses; **h, i**. Columellae with collars and projections; **j**. Rhizoids; **k–m**. Chlamydospores; **n**. Sporangiospores. Scale bars: 10 µm (**c–n**).

##### Holotype.

HMAS 354601.

##### Description.

Colonies on PDA at 25 °C for 9 days, growing slowly, attaining 61–65 mm in diameter, showing an average growth rate of approximately 6.8–7.2 mm/d, initially white, velvety, irregularly and concentrically zonate with a ring, and radially in reverse. Hyphae branched, subhyaline, light grayish, and 3.8–7.7 µm in diameter. Stolons subhyaline, gradually becoming brownish when mature, smooth, branched, some septate, and 2.9–6.5 µm in diameter. Rhizoids abundant, root-like, branched, and subhyaline. Sporangiophores arising from stolons, smooth, mostly erect, unbranched or simply branched, monopodial or sympodial, some 2–4 in whorls, subhyaline, brownish when mature, often with a septum 2.2–4.0 µm below the apophysis, 35.9–171.5 µm long, and 2.5–4.4 µm wide. Sporangia pyriform, smooth, subhyaline, deliquescent-walled, multi-spored, 18.5–18.9 µm long, and 15.3–17.4 µm wide. Apophyses distinct, subhyaline, 3.2–7.8 µm high, gradually widening from the base to the top, 2.2–4.9 µm wide at the base, and 6.0–9.3 µm wide at the top. Collars present. Columellae subglobose to globose, subhyaline, 6.5–11.5 µm long, and 6.6–10.8 µm wide. Projections present, single, pacifier-like, subhyaline, 3.9–9.5 µm long, and 1.0–2.4 µm wide. Sporangiospores usually cylindrical, sage green, smooth, 3.3–3.7 µm long, and 1.4–2.2 µm wide. Chlamydospores present, terminal or intercalary, and thick-walled. Zygospores not observed.

##### Materials examined.

China • Guangxi Zhuang Autonomous Region, Chongzuo City, Jiangzhou District, Provincial Highway 313, Chongzuo Ecological Park (22°16.64'N, 107°30.77'E), in the soil, 28 November 2024, Heng Zhao (holotype HMAS 354601, ex-holotype living culture CGMCC 3.29876 = XG21645-1-1).

##### GenBank accession numbers.

ITSPZ378781; LSUPZ381891; SSUPZ385219; *act*PZ392928; *TEF1α*PZ414687.

##### Notes.

In the phylogenetic analyses, based on the concatenated ITS-LSU-*TEF1α*-*act*-SSU sequences, the newly identified strains of *Absidia myceliosa* sp.nov. are intimately allied to the aforementioned new species *A. longisporangiophora* (Fig. 1: MLBS = 100%, BIPP = 1). The pairwise identity values between *A. myceliosa* and *A. longisporangiophora* are 95.6% for ITS, 98.9% for LSU, 98.6% for *TEF1α*, 99.4% for *act*, and 99.8% for SSU. The previous notes of *A. longisporangiophora* provide the morphological differences between the two new species.

#### Gongronella globospora

Taxon classificationFungiMucoralesCunninghamellaceae

F. Li, Z.M. Fang & X.Y. Liu sp. nov.

0C92D6CA-EDAD-5C51-92CE-8219D6657806

Fungal Names: FN 573950

Fig. 8

##### Etymology.

The epithet *globospora* (Lat.), refers to the globose sporangia.

**Figure 8.**
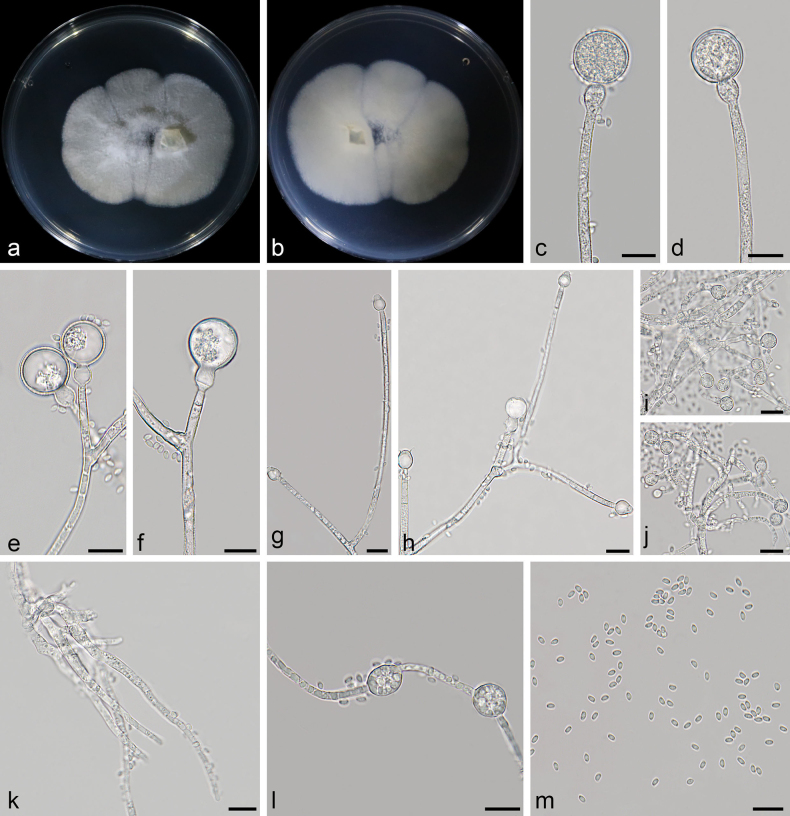
Morphologies of *Gongronella globospora* (HMAS 354602, holotype). **a, b**. Colonies on PDA (**a**. Obverse; **b**. Reverse); **c, d**. Unbranched sporangiophores with mature sporangia; **e, f**. Unbranched sporangiophores with sterile sporangia; **g, h**. Branched sporangiophores with columellae, collars, apophyses, septa; **i, j**. Giant cells; **k**. Rhizoids; **l**. Intercalary chlamydospores; **m**. Sporangiospores. Scale bars: 10 µm (**c–m**).

##### Holotype.

HMAS 354602.

##### Description.

Colonies on PDA in the dark at 25 °C for 8 days, growing moderately, attaining 46–62 mm in diameter, exhibiting an average growth rate of approximately 5.8–7.8 mm/d, white throughout the entire growth phase, cottony on the colony’s surface and irregularly notched at the edge, and milky white in reverse. Rhizoids root-like, subhyaline, branched, and contain oil droplets. Stolons absent. Sporangiophores arising from aerial hyphae, mostly subhyaline, smooth, erect or bent, unbranched or branched 2–4 times, 28.3–175.2 µm long and 1.8–3.8 µm wide, usually with one septum 2.2–3.3 µm below the apophysis, and partly containing oil droplets. Sterile sporangia present, hyaline, globose or rarely gourd-shaped, 7.7–15.9 µm in diameter. Fertile sporangia light brown, subhyaline, globose, smooth, deliquescent-walled, 15.3–16.6 µm in diameter, and sometimes leaving collars when sporangiospores are released. Columellae hyaline, subglobose, smooth, 1.8–5.2 µm long and 1.1–3.3 µm wide. Apophyses mostly hyaline, smooth, ellipsoidal, 6.5–8.4 µm long and 6.0–6.9 µm wide. Giant cells present, subhyaline, globose, and 3.7–7.4 µm in diameter. Sporangiospores light green or sage green, smooth, diamond to oval, 1.9–2.9 µm long and 1.3–1.8 µm wide. Chlamydospores present, subhyaline, ellipsoidal to globose, 7.2–18.8 µm in diameter, terminal or intercalary, and filling with oil droplets. Zygospores absent.

##### Materials examined.

China • Fujian Province, Sanming City, Sanyuan District, Sanyuan National Forest Park, Geshi Oak Scenic Area, 120 meters northeast to the Visitor Center (26°10.28'N, 117°27.72'E), in the soil, 24 October 2024, Yang Jiang (holotype HMAS 354602, ex-holotype living culture CGMCC 3.29877 = XG12236-12-1); China • Fujian Province, Sanming City, Sanyuan District, Sanyuan National Forest Park, Geshi Oak Scenic Area, 120 meters northeast to the Visitor Center (26°10.28'N, 117°27.72'E), in the soil, 24 October 2024, Yang Jiang, living culture XG12246-12.

##### GenBank accession numbers.

ITSPV491274; LSUPV603470; *act*PZ433251; *TEF1α*PZ433253.

##### Notes.

In the phylogenetic analyses, based on the concatenated ITS-LSU-*act*-*TEF1α* sequences, the newly identified strains of *G. globospora* sp. nov. are closely related to *G. chlamydospora* (Fig. 2: MLBS = 100%, BIPP = 1). The pairwise identity values between *G. globospora* and *G. chlamydospora* are 93.4% for ITS. The values of LSU, *act*, and *TEF1α* are not available. Morphologically, they are differentiated in growth rate (5.8–7.8 mm/d vs. 8.2 mm/d), apophyses (6.5–8.4 × 6.0–6.9 µm vs. 6.0–12.0 × 6.0–10.0 µm), columellae (1.8–5.2 × 1.1–3.3 µm vs. 3.0–5.5 × 3.5–6.5 µm) and chlamydospores (7.2–18.8 µm vs. 2.0–3.0 µm) (Zhao et al. 2023).

## Discussion

Cunninghamellaceae is widely distributed in subtropical regions and occurs on multiple substrates, including soil and compost heaps (Lima et al. 2018; Zong et al. 2021). The six new species described in this article were isolated from soil samples collected in the temperate and subtropical zones. The soil samples were collected from six different areas in China: Anhui Province, Fujian Province, Guangdong Province, Guangxi Zhuang Autonomous Region, Heilongjiang Province, and Hubei Province. These six regions have completely different natural conditions. The Anhui Province has a transitional climate between warm temperate and subtropical zones, characterized by mild temperatures, moderate precipitation, and clear seasons; Fujian Province has a subtropical maritime monsoon climate, which is almost always warm and humid; Guangdong Province has a subtropical monsoon climate, which has high temperatures and abundant rainfall throughout the year; Guangxi Zhuang Autonomous Region has a subtropical monsoon climate, which is warm and humid all year; Heilongjiang Province has a cold-temperate to temperate continental monsoon climate with a large annual temperature range, dry winters, and rainy summers; Hubei Province has a subtropical monsoonal humid climate, which is characterized by four distinct seasons and abundant precipitation. The soil types of Anhui Province are diverse, exhibiting an obvious transitional characteristic from north to south, containing yellow-cinnamon soil, red soil, and so on; Fujian Province are various, including red soils, yellowish soils and coastal solonchak; Guangdong Province mainly has red soil, lateritic red soil, and laterite, characterized by strong acidity and low nutrient content; Guangxi Zhuang Autonomous Region is mainly acidic red soils enriched in iron and aluminum; Heilongjiang Province is characterized by fertile black soils and dark brown soils; and Hubei Province has diverse soil types, including yellow brown soils, yellow cinnamon soils, and so on. The various natural conditions in China, including climate and soil, support numerous microbial resources.

In this study, we proposed six new species of two genera in Cunninghamellaceae: *Absidia guangxiensis* sp. nov., *A. heilongjiangensis* sp. nov., *A. hubeiensis* sp. nov., *A. longisporangiophora* sp. nov., *A. myceliosa* sp. nov., and *Gongronella globospora* sp. nov. They were identified through combining morphological observation and phylogenetic analyses. Molecular phylogeny of *Absidia* showed that *A. guangxiensis* sp. nov. and *A. heilongjiangensis* sp. nov. were sister clades, forming an independent clade with high support (Fig. 1: MLBS = 99%, BIPP = 1); *A. hubeiensis* sp. nov. was related to *A. cylindrospora* (Fig. 1: MLBS = 89%, BIPP = 0.99) (Ho et al. 2004); *A. longisporangiophora* sp. nov., and *A. myceliosa* sp. nov. were sister clades and also formed a full-supported independent clade (Fig. 1: MLBS = 100%, BIPP = 1). Phylogenetic analyses of *Gongronella* exhibited that *G. globospora* sp. nov. was allied to *G. chlamydospora* with full support (Fig. 2: MLBS = 100%, BIPP = 1). Morphological analyses indicated that these six new species differed significantly from their sister clades in growth rate, the length and width of hyphae, stolons, and sporangiophores, the size of chlamydospores, sporangia, columellae, and sporangiospores, and the volume of apophyses and projections.

Species of the family Cunninghamellaceae have significant value in industry. In the genus *Absidia*, for example, *A. repens* can produce chitosan that is widely used in industrial and medical fields (Davoust and Persson 1992; Kaczmarek et al. 2019); *A. reflexa* can secrete α-galactosidase as an enzymatic catalyst to synthesize rubusoside derivatives (Kitahata et al. 1989; Zhao et al. 2022); and *A. spinosa* can secrete laccase to induce biotransformate (Kristanti et al. 2016). The genus *Gongronella* also exhibits high potential for biological applications, for example, *G. butleri* yields high amounts of chitosan and is also commonly used in industry (Tan et al. 1996; Babu et al. 2015); *G. butleri* w5 regulates carbon allocation by the sucrose transporter protein GspSUT1, which enhances the activation of nitrogen-fixing bacteria, increases soil nitrogen content, and promotes plant growth (Fang et al. 2025); and *G. butleri* w5 also releases aromatic volatile organic compounds such as 1H-indole-4-carboxaldehyde and 4-methylbenzyl alcohol to promote the development of plant roots when co-cultured with plants (Fang et al. 2026).

Therefore, future research on Cunninghamellaceae should integrate morphological and molecular phylogenetic analyses to better reveal the species richness of this group, clarify its taxonomic placement, and resolve evolutionary relationships of the zygomycotan fungi. Furthermore, genomic predictions and explorations could enhance the application value of Cunninghamellaceae in industry, medicine, and other fields.

The six newly proposed species in this study bring the number of accepted species of *Absidia* and *Gongronella* to 71 and 34, respectively, increasing the biodiversity of the family Cunninghamellaceae in China. This paves the way for exploring these applications in the future.

## Supplementary Material

XML Treatment for Absidia guangxiensis

XML Treatment for Absidia heilongjiangensis

XML Treatment for Absidia hubeiensis

XML Treatment for Absidia longisporangiophora

XML Treatment for Absidia myceliosa

XML Treatment for Gongronella globospora

## Additional information

### Artificial Intelligence (AI) use

The authors accept full responsibility for the content of the manuscript, including the disclosure of any use of AI.

No AI tools were used in the preparation of this manuscript.

### Author ORCIDs

F. Li https://orcid.org/0009-0002-7969-6671

R.-P. Zhang https://orcid.org/0000-0001-6050-513X

J.-N. Fang https://orcid.org/0009-0000-4463-6885

X.-J. Wang https://orcid.org/0000-0002-8102-4658

H.-Y. Zou https://orcid.org/0009-0005-6469-4667

S.-T. Geng https://orcid.org/0009-0005-4074-0657

S. Wang https://orcid.org/0000-0002-7376-7638

Z.-M. Fang https://orcid.org/0000-0002-9853-5470

X.-Y. Liu https://orcid.org/0000-0002-8808-010X

### Data availability

All the data that support the findings of this study are available in the main text. The sequences were deposited in the GenBank database.

## Supplementary materials

Supplementary material 1 GenBank accession numbers Data type xlsx Explanation note Taxa information and corresponding GenBank accession numbers used in this study. https://binary.pensoft.net/file/1711213This dataset is made available under the Open Database License (http://opendatacommons.org/licenses/odbl/1.0/). The Open Database License (ODbL) is a license agreement intended to allow users to freely share, modify, and use this Dataset while maintaining this same freedom for others, provided that the original source and author(s) are credited. Fei Li, Ri-Peng Zhang, Jun-Nan Fang, Xiao-Jie Wang, Hong-Yu Zou, Shu-Ting Geng, Shi Wang, Ze-Min Fang, Xiao-Yong Liu
